# Hyperbaric oxygen therapy as a potential treatment for MDD: a narrative review

**DOI:** 10.3389/fmed.2026.1931426

**Published:** 2026-10-01

**Authors:** Yun Chen Pang, Peter Giacobbe, Fahad Alam

**Affiliations:** 1 Institute of Medical Science, Temerty Faculty of Medicine, University of Toronto, Toronto, ON, Canada; 2 Department of Psychiatry, Temerty Faculty of Medicine, University of Toronto, Toronto, ON, Canada; 3 Harquail Centre for Neuromodulation, Sunnybrook Health Sciences Centre, Toronto, ON, Canada; 4 Kerr Chair in Interventional Psychiatry, Sunnybrook Health Sciences Centre, Toronto, ON, Canada; 5 Department of Anesthesia, Sunnybrook Health Sciences Centre, Toronto, ON, Canada; 6 Department of Anesthesiology and Pain Medicine, University of Toronto, Toronto, ON, Canada; 7 School of Medicine, Toronto Metropolitan University, Toronto, ON, Canada

**Keywords:** antidepressant, anti-inflammatory, depression, hyperbaric oxygen therapy, posttraumatic stress disorder, stroke

## Abstract

This narrative review explores the therapeutic potential of hyperbaric oxygen therapy (HBO_2_) in treating major depressive disorder (MDD). We highlight the common pathophysiological hypotheses for MDD, the strengths and limitations of current MDD treatments, and the potential for HBO_2_ to be a novel MDD therapeutic through anti-inflammatory and neuroprotective mechanisms. The current review discusses the monoamine, neuronal atrophy, and inflammation hypotheses of MDD pathophysiology origin, as well as how present MDD therapeutics target these MDD pathophysiological traits. While there are a multitude of existing treatments for MDD, including those pharmacological, psychotherapeutic, and neuromoduatory in nature, many treatments possess limitations. The management of difficult-to-treat depression is particularly challenging, highlighting the need for innovative, effective treatments to be developed. HBO_2_ is a potential novel therapy for MDD, exerting antidepressant effects through its ability to promote neurogenesis and anti-inflammatory processes in the central nervous system. Moreover, HBO_2_ has been indirectly correlated with an improvement in depressive symptoms in studies done in the context of treatment for post-traumatic stress disorder (PTSD), traumatic brain injury (TBI), post-stroke recovery, and post-coronavirus disease condition (post-COVID-19). However, there is currently a dearth of direct evidence supporting the indication of HBO_2_ for MDD, and clinical trials that investigate HBO_2_ as a primary treatment for MDD are needed. The current review provides a rationale for further exploration of the potential of HBO_2_ as a novel therapy for MDD.

## 1 Literature search strategy

From January 2026 to July 2026, we searched through the following databases for literature related to hyperbarics and MDD: MEDLINE (Ovid), Google, PubMed, and Web of Science (Clarivate). The following search terms were used: “major depressive disorder”, “depression”, “MDD”, “monoamine”, “neuronal atrophy”, “neuroinflammation”, “psychotherapy”, “antidepressant”, “selective serotonin reuptake inhibitors”, “SSRI”, “DTD”, “difficult to treat depression”, “TRD”, “treatment resistant depression”, “neuromodulation”, “brain stimulation”, “rTMS”, “repetitive transcranial magnetic stimulation”, “ECT”, “electroconvulsive therapy”, “transcranial direct current stimulation”, “tDCS”, “magnetic seizure therapy”, “MST”, “deep brain stimulation”, “DBS”, “glutamate modulator”, “ketamine”, “psychedelics”, “hyperbaric oxygen therapy”, “HBOT”, “HBO”, “PTSD”, “post traumatic stress disorder”, “COVID-19”, “post-COVID-19”, “post stroke depression”, “PSD”, “traumatic brain injury”, “TBI”. These key words were cross-referenced with key words such as “efficacy”, “prevalence”, “therapy”, “therapeutic”, “treatment”, etc. We prioritized systematic reviews and meta-analyses, randomized controlled trials, but did not exclude any sources of data, including data from public government sources.

## 2 Introduction

Major depressive disorder (MDD) is a debilitating mood disorder with a one-year prevalence of 4.4% and a lifetime prevalence ranging from 2% - 20% worldwide (1, 2). The global disease burden of MDD has seen a persistent upward trajectory, demonstrated by a growth in prevalence from 170 million people in 1990 to 280 million people in 2019 (3). In addition to the widespread prevalence of MDD, the disorder also entails a significant health and socioeconomic burden to individuals affected and the healthcare system. Depression ranks 13th in disease adjusted life years (DALYs) and 2nd in years lost to disease (YLD) globally (3).

Although multiple evidence-based treatments for MDD exist, there is an urgent need to develop novel therapeutics to circumvent limitations of existing treatments, as well as increase available therapeutic options to reduce the mortality and morbidity associated with this condition (4). This narrative review will discuss existing hypotheses for MDD pathophysiology and current treatments, as well as the potential for hyperbaric oxygen therapy (HBO_2_), a treatment traditionally known for its anti-inflammatory effects, to treat MDD. HBO_2_ is a treatment that utilizes a pressurized chamber and medical-grade high concentration oxygen for a wide variety of indications, such as wound healing, stroke, carbon monoxide poisoning, etc. While HBO_2_ is not indicated as a treatment for any psychiatric disorders at the present time, existing studies have observed reductions in psychiatric symptoms, including depression, after HBO_2_ treatment. The present review will discuss these findings, as well as potential mechanisms of actions by which HBO_2_ could treat MDD.

## 3 Pathophysiology of MDD: guiding novel therapeutics

MDD is a mood disorder characterized by a diverse array of psychological, cognitive and neurovegetative symptoms centered around the core feature of low mood or anhedonia occurring consistently for at least two weeks (5, 6). Due to the heterogeneity of the condition, multiple hypotheses exist explaining the pathophysiology of MDD, including the monoamine, the neurotrophic, and neuroinflammation hypotheses (7).

### 3.1 Monoamine hypothesis

One of the most commonly described explanatory model of MDD is the monoamine hypothesis, which suggests that depressive symptoms are secondary to deficiencies in monoamine neurotransmitters, the two most common ones being serotonin and norepinephrine (8). In this model, selective serotonin reuptake inhibitors (SSRIs), a class of medications that have been widely used as a treatment for depression, increase the amount of serotonin available in the synaptic cleft to address this hypoactivity (8). However, unresolved questions from the monoamine hypothesis includes the fact that SSRIs have been shown to inhibit serotonin transporters within hours, yet the antidepressant effects of SSRIs treatment require 4–8 weeks to fully manifest (8). Furthermore, due to the presence of serotonin receptors throughout the body, the lack of anatomical specificity of SSRIs could lead to multiple side effects, such as nausea, sexual dysfunction, and gastrointestinal issues (9). Moreover, around one third of MDD patients are nonresponsive to SSRI treatment, highlighting that pharmacological modulation of monoamines is not a comprehensive treatment strategy for MDD (10).

### 3.2 Neuronal atrophy hypothesis

An alternative hypothesis suggests that MDD pathology could be a result of neuronal atrophy from deficiencies in brain-derived neurotrophic factor (BDNF) (11). Brain imaging studies have revealed that MDD patients experience a reduction in grey matter volume in the prefrontal cortex (PFC) and hippocampus, and post-mortem studies indicate that antidepressants increase BDNF concentration in the brain (11, 12), suggesting that treatments for MDD can be neurorestorative. A pivotal line of evidence supporting the neurotrophic hypothesis originates from literature demonstrating local infusions of BDNF in behavioral mouse models inducing an antidepressant effect (13). However, studies examining the effect of BDNF depletion on MDD symptoms show mixed results, with the effects varying depending on the knockout and behavioral methodology (11).

### 3.3 Neuroinflammation hypothesis

In addition to the monoamine hypothesis and neurotrophic hypothesis, it has also been hypothesized that neuroinflammation may contribute to the pathophysiology of MDD (7). The basis of this hypothesis lies in the cascade of downstream effects induced by neuroinflammation in the central nervous system (CNS). Under neuroinflammatory conditions, the metabolic branch of the kynurenine pathway that produces tryptophan (TRP), a molecule essential for producing serotonin, is inhibited. At the same time, the alternative metabolic branch of the kynurenine pathway producing quinolinic acid, a molecule associated with neurotoxicity and excitotoxicity, is activated, leading to neuronal damage and apoptosis (14). Another effect of chronic neuroinflammation is reduced glucocorticoid sensitivity (15). Under normal circumstances, when neuroinflammation or stress occurs, glucocorticoids are released to signal anti-inflammatory responses (15). However, intense, sustained stress-induced chronic glucocorticoid release results in desensitization of glucocorticoid receptors (15). This leads to a sustained elevation in cortisol levels, which in turn leads to hippocampal atrophy (15). Reactive oxygen species (ROS) concentrations are also increased under neuroinflammatory conditions, leading to neuronal damage and toxicity (16). While some studies have investigated the use of anti-inflammatory agents such as nonsteroidal anti-inflammatory drugs (NSAIDS) to treat MDD, the safety of combining anti-inflammatory agents with antidepressants, as well as the potential side effects of multiple anti-inflammatory agents have been major areas of concern (17). Nevertheless, developing treatments that directly target inflammatory processes in the brain is a novel approach to tackling MDD, and identifying new anti-inflammatory therapies that are lower in risk could increase therapeutic options for patients.

## 4 Current treatments for depression and their limitations

Clinical management and treatment of MDD can vary depending on the severity of the symptoms and patient preference. Psychotherapy, pharmacotherapy utilizing selective serotonin reuptake inhibitors (SSRIs), and a combination thereof are considered 1st line treatments for mild to moderate MDD with minimal safety risk (18, 19). In cases where patients do not respond to therapeutic dosages of psychotherapy and SSRIs, or where symptoms are severe and a high patient safety risk is present, other treatments such as neuromodulation or ketamine may be more appropriate (5, 7, 19).

### 4.1 Psychotherapy and first-line medications

Psychotherapy, or talk therapy, describes an interactive treatment involving patients engaging in therapeutic conversations with licensed mental health professionals (20). Common types of psychotherapy used clinically include cognitive behavioral therapy (CBT), interpersonal therapy (IPT), and behavioral activation therapy (BAT) (19, 21). Psychotherapy is unique in that although it is a broad categorization of many different forms and subcategories of talk-based therapy, the efficacies of different therapies do not differ significantly from one another (22). Evidence shows that when compared to controls, psychotherapies are more effective at reducing MDD symptoms, with this higher efficacy observed consistently across a wide variety of psychotherapies (23).

As described in the Section 3.1, SSRIs aim to increase the serotonin concentration at the synaptic cleft, potentially leading to an improvement in depressive symptoms. Many professional psychiatric organizations recommend SSRIs as a first-line treatment for MDD, and meta-analyses on long-term placebo-controlled SSRI efficacy trials indicate SSRI treatment yields approximately a 20% increase in response rate compared to controls (24, 25).

Despite recommended as first-line treatments for MDD, SSRI-based pharmacotherapy and psychotherapy are not without limitations. SSRIs have many potential side-effects, including gastrointestinal issues, weight gain, sexual side effects, and dry mouth (19). Accessibility and adherence to treatment are also of concern, with approximately 30%–50% of MDD patients prescribed with SSRIs not adhering to the acute phase of treatment (26). Psychotherapy is limited in its accessibility due to the availability of professional psychotherapy providers, facility requirements, financial costs, and time commitments from patients, although virtual forms of psychotherapy are aiming to tackle these limitations (27). Furthermore, despite SSRIs and psychotherapy having demonstrated superior efficacy compared to controls, response rates to these treatments are often limited to approximately 60% of cases (22, 25). Approximately one third of MDD patients have what is called treatment resistant depression (TRD) or difficult-to-treat depression (DTD), which is when the patient does not respond to first-line SSRI and psychotherapy treatments (19, 28). Patients with DTD require adjunctive treatment approaches in order to alleviate their symptoms and potentially achieve remission.

### 4.2 Neuromodulation

Neuromodulation, or brain stimulation, is an established approach to treat DTD (19, 29). Standard neuromodulation therapies include repetitive transcranial magnetic stimulation (rTMS) and electroconvulsive therapy (ECT), with alternative neuromodulation methods such as transcranial direct current stimulation (tDCS), magnetic seizure therapy (MST), and deep brain stimulation (DBS) prescribed at lower frequencies (30).

rTMS is a non-invasive neuromodulation treatment that utilizes electromagnetic induction to produce transient electrical currents in underlying cortical tissue, either to stimulate or inhibit brain activity, depending on the stimulation intensity and frequency (31). While rTMS is less stigmatized and has relatively milder side effects compared to other, more invasive neuromodulation treatments, its relatively lower remission rates and accessibility issues limit its effectiveness as a ubiquitous treatment for DTD. Remission rates after rTMS treatment can range from 30% – 60% (29, 32).

ECT involves the induction of a short general seizure in an anesthetized patient via electrodes placed on the scalp (19). ECT has the highest efficacy rate compared to other neuromodulation treatments, and remission rates after ECT treatment are reported to be approximately 60% (29, 32). Although ECT is efficacious and safe, its rate of use may be limited by stigma, as well as cognitive and memory side effects, which are major concerns for some patients (33–35).

Compared to the previously mentioned treatments, DBS is unique in that it is an invasive, surgical procedure where electrodes are placed intracranially to constantly deliver stimulation via a pulse generator also surgically placed in the patient's chest wall (29). Although DBS has demonstrated promising results with long-term open-label stimulation for those with extreme forms of DTD, evidence of superiority of active versus sham stimulation in double-blinded randomized control trials is still lacking (36). The limitations of DBS predominantly lie in the invasiveness of the procedure and risk for complications (29). Similar to ECT, DBS is often stigmatized as a harmful and personality-altering procedure despite being an efficacious neuromodulatory treatment (37).

### 4.3 Glutamate modulators and psychedelics

Glutamate modulators, particularly ketamine, have been growing as a potential group of pharmacological antidepressants alternative to SSRIs (5). When comparing efficacy rates between ketamine and ECT, the current gold-standard treatment for DTD, Anand et al. found that ketamine was comparable to ECT in terms of efficacy in MDD symptom reduction for DTD patients without psychosis (38). One of the significant strengths of ketamine is its rapid antidepressant and anti-suicidality effects, ranging from 2 to 4 h post infusion (39). Ketamine is typically dosed at 0.5–1.0 mg/kg of body weight, and either twice a week or three times a week (38, 40). Currently the administration routes for ketamine with the most robust evidence are intravenous and intranasal (19). While the rapid antidepressant effect and efficacy of ketamine is promising, it is not without limitations. The literature has shown that ketamine only induces an antidepressant response in around 45% of patients, with around only 30% of patients achieving remission (41). Furthermore, there is currently a paucity of evidence regarding baseline clinical predictors of response (42). The short half-life of ketamine also means that its antidepressant effects can be transient, with studies showing antidepressant effects lasting around a week after infusion (39). Finally, side effects including dissociation and the costs of the treatments limit the tolerability and accessibility of ketamine (19, 38).

In summary, although a wide variety of therapeutic options exist for MDD, issues around their accessibility, acceptability, side-effect profiles and lack of complete response, have prompted the need for further exploration of novel approaches to ameliorate depressive symptoms in those who do not respond to established strategies.

## 5 Hyperbaric oxygen therapy: introduction to mechanisms and potential as an MDD therapeutic

HBO_2_ is a treatment that places patients a pressurized chamber higher than ambiance pressure, while breathing medical-grade oxygen, which is oxygen that has greater than 99% oxygen purity (43). The Undersea & Hyperbaric Medical Society (UHMS) has identified 15 indications for which scientific evidence exists supporting HBO_2_ use in treatment. These indications include: Air or gas embolism, carbon monoxide poisoning, carbon monoxide poisoning complicated by cyanide poisoning, clostridial myositis and myonecrosis, acute traumatic ischemia, decompression sickness, central retinal artery occlusion, wound healing, severe anemia, intracranial abscess, necrotizing soft tissue infections, osteomyelitis, delayed radiation therapy, compromised grafts and flaps, acute thermal brain injury, idiopathic sudden sensorineural hearing loss, and avascular necrosis (44). In addition, studies have examined the use of HBO_2_ in treating neurological conditions, such as traumatic brain injury (TBI) and stroke (45). HBO_2_ has demonstrated promising neuroprotective effects following a stroke, reducing inflammation in the CNS and promoting neuroplasticity (45).

### 5.1 Potential mechanisms of action

The potential mechanisms by which HBO_2_ improve brain health are likely derived directly through increasing plasma oxygen concentration and inducing anti-inflammatory effects within the CNS (46). HBO_2_ is indicated for the treatment of various brain injury conditions, largely due to the recovery and treatment of these conditions requiring additional oxygen on top of the already high oxygen consumption of the brain at a healthy state (47). Indeed, evidence suggests that HBO_2_ hyperoxygenates cerebral blood flow, providing a surplus of oxygen for aerobic cellular respiration and energy production, and reducing cellular apoptosis (46, 47). At the tissue level, previous literature examining the effects of HBO_2_ in treating carbon monoxide poisoning have found white matter repair to occur after the treatment (48). HBO_2_ has also been shown to increase the synthesis of reactive oxygen species (ROS) (45). While ROS is typically associated with brain injury and toxicity, short-term production of ROS during HBO_2_-induced hyperoxia has been shown to stimulate the synthesis of growth factors, initiate immunomodulatory effects, and reduce neovascularization in stroke patients (45).

In addition to increased neuroplasticity and wound repair, HBO_2_ has also demonstrated anti-inflammatory effects. In a rat cerebral ischemia model, HBO_2_ treatments at both 4 and 12 h saw a significant downregulation of pro-inflammatory factors tumor necrosis factor alpha and interleukin 1-beta (49). In animal irritable bowel disease models, HBO_2_ has also been shown to reduce pro-inflammatory cytokine release, suggesting that HBO_2_ elicits a systemic anti-inflammatory effect across multiple organ systems (50). Pre-treatment of neuronal cell models with HBO_2_, followed by introducing inflammatory damage has revealed HBO_2_-treated cells to have greater viability, likely due to HBO_2_ inducing mitochondria transfer prior to inflammatory insult (51). This indicates that HBO_2_ potentially has both protective and alleviating effects against inflammatory conditions, which could counteract the chronic inflammation hypothesized to occur in MDD (52).

At a neuroanatomical level, brain perfusion studies have revealed HBO_2_ treatment in fibromyalgia patients led to an increase in blood flow in the dorsolateral prefrontal cortex (DLPFC), ventrolateral prefrontal cortex (VLPFC), and hippocampus (53). These effects could potentially be leveraged for addressing the maladaptive activity changes that occur between brain structures in MDD, including decreased activity in the DLPFC and VLPFC (54). HBO_2_ has also demonstrated therapeutic effects in conditions where resting state functional connectivity between neural networks is disrupted. In a study assessing HBO_2_ treatment for post-coronavirus disease condition (post-COVID-19), HBO_2_ treatment was found to reverse hyperconnectivity within the default mode network (DMN) and increase connectivity between the DMN and salience network (SN) (55). The abnormal upregulation of DMN activity and decreased connectivity between the DMN and SN are consistently observed in MDD patients, and the observations of HBO_2_ reverting these maladaptive connectivity changes back to homeostasis in post-COVID-19 patients suggest potential for therapeutic use in MDD patients as well (56). As these potential mechanisms by which HBO_2_ could have a therapeutic effect towards MDD are largely theoretical, research assessing the effects of HBO_2_ on validated MDD models and MDD patients is necessary to determine the applicability of HBO_2_ to treat the maladaptive brain activity symptoms observed in MDD. Figure 1 organizes the proposed mechanisms of action by which HBO2 could influence brain physiology, and subsequently improving brain health (Figure 1).

**Figure 1:**
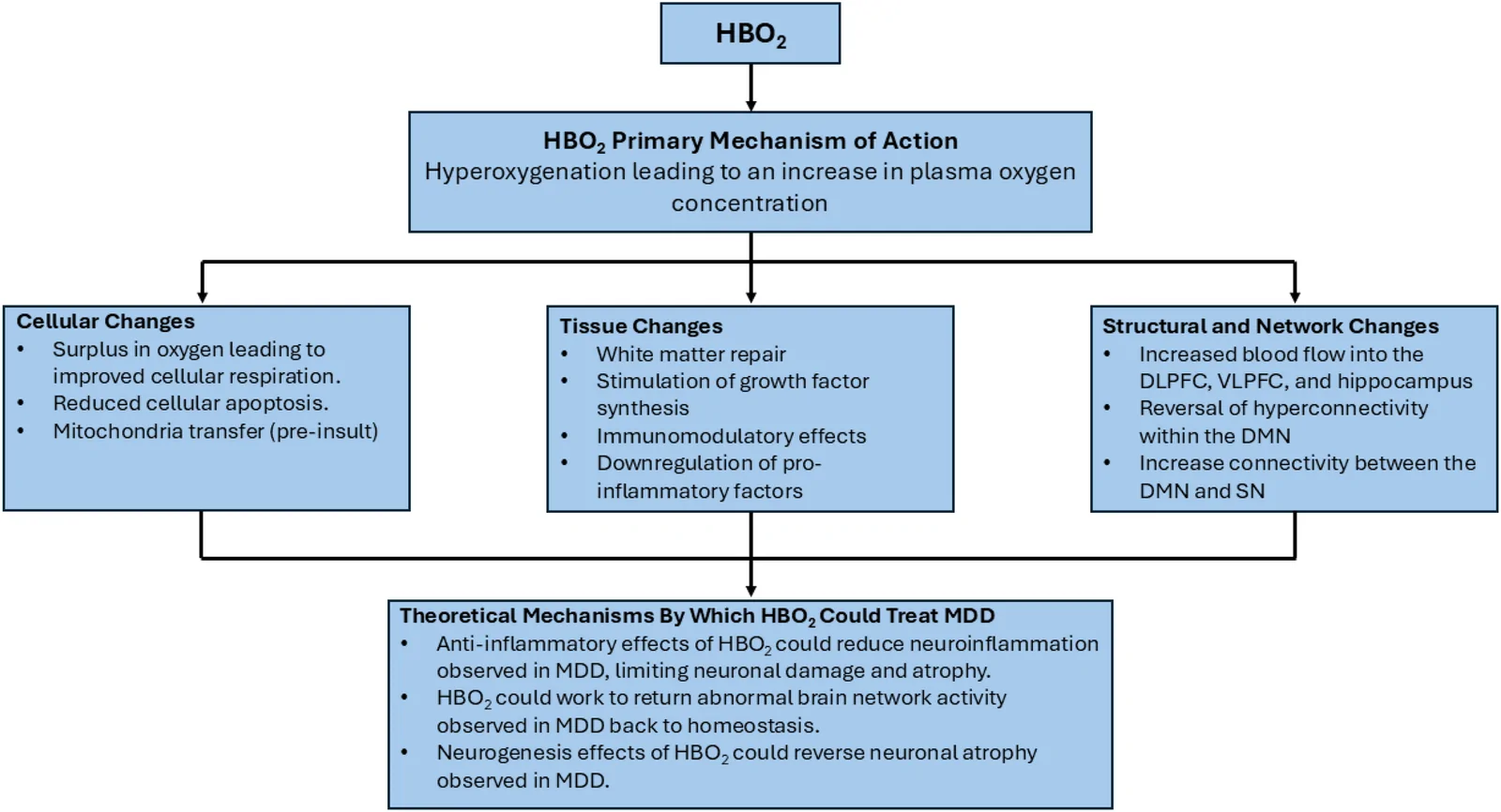
Schematic of theoretical mechanisms of HBO_2_ treatment for MDD. The schematic summarizes the discussion in the previous section postulating potential mechanisms of action by which HBO_2_ could influence brain physiology and produce an antidepressant effect. Note that the ideas presented in the figure are largely theoretical, and more research assessing the direct effect of HBO_2_ in an MDD cohort is needed.

### 5.2 Evidence of HBO_2_ effects on psychiatric symptoms in neuropsychiatric conditions and brain injury

Although the use of HBO_2_ to treat MDD directly has yet to be explored, there is an emerging body of literature in its potential to treat psychiatric disorders. Over the past decade interest in HBO_2_ use for post-traumatic stress disorder (PTSD) and traumatic brain injury (TBI) has been growing, with preliminary studies demonstrating symptomatic improvements in these neuropsychiatric conditions (57–59). While the main objective of these studies was to examine the therapeutic effects of HBO_2_ for PTSD and TBI, findings have also been made on the effects of HBO_2_ toward alleviating anxiety and depressive symptoms in their secondary outcomes.

One common symptom that many HBO_2_ studies for PTSD examine is depressive symptoms. Multiple HBO_2_ studies on PTSD patients have shown noticeable improvements in depressive and occasionally anxiety symptoms after treatment. In Doenyas-Barak et al's study assessing the neurocognitive and brain structure changes HBO_2_ induces in a veteran population with PTSD, the group observed a 6.3 score or a 25.8% decrease on the Beck depression inventory-II (BDI-II) questionnaire in the HBO_2_ treatment group after treatment (57). This exceeds the minimal clinically important difference (MCID) of the BDI-II questionnaire, which is 17.5%, indicating that participants receiving HBO_2_ in this study experienced a clinically significant decrease in depressive symptoms (60). Similarly, Harch et al's study on utilizing HBO_2_ for TBI and PTSD patients found an 8.4 score decrease in the Patient Health Questionnaire-9 (PHQ-9) scores (61). However, these findings were secondary outcomes of the studies, and it is unclear whether the reduction in depressive symptoms is due to the direct effect of HBO_2_, or due to alleviation of PTSD and TBI symptoms. In addition to PTSD, studies looking at using HBO_2_ to treat spinal cord injury (SCI), the HBO_2_ treatment groups have demonstrated similar improvements in depressive and anxiety symptoms compared to controls (62). In a study comparing the effects of HBO_2_ and psychotherapy on improving depressive and anxiety symptoms for SCI patients, Feng et al. found the effects of HBO_2_ to be similar to that of psychotherapy in reducing depression and even surpassing psychotherapy in reducing anxiety (63). While these results are promising, it is unclear if these significant improvements in psychological symptoms were due to a direct effect of HBO_2_ on MDD pathways, or due to an improvement in SCI symptoms, which were observed in the study. Furthermore, the average Hamilton Depression Rating Scale (HAMD) scores of participants before treatment were around 17, which is the minimum score to be considered for moderate MDD (64). Thus, it is unclear if HBO_2_ will produce similar improvements in psychological symptoms for patients with more severe MDD.

In a similar study, Sun et al. investigated the effect of HBO_2_ in improving the limb function and mood of cerebral infarction (CI) patients that were paralyzed on one side of their bodies (65). They found that when combined with nursing care optimizations, HBO_2_ treatment has shown to improve neurological function and decrease depression and anxiety in cerebral infarction patients (65). Interestingly, in a study examining the effect of HBO_2_ in alleviating post stroke depression (PSD), Guo et al. believe that HBO_2_ was able to increase blood supply to lesions from stroke and improve neurological function, potentially explaining the improvement of depressive symptoms observed (66). This observation aligns with what was found in a meta-analysis assessing the effect of HBO_2_ on PSD across 27 randomized controlled trials (RCT). In the meta-analysis, HBO_2_ treatment groups saw greater reductions in scales examining MDD severity, such as the HAMD, as well as improvement to other indicators such as immobility in rats (67). Previously, it was discussed that HBO_2_ has demonstrated therapeutic effects in regulating abnormalities in brain network connectivity in post-COVID-19, which is similar to the abnormalities observed in MDD (55, 56). Another study done by Zilberman-Itskovich et al. provides further indirect support for HBO_2_ use in treating depressive symptoms by demonstrating HBO_2_ to improve psychiatric symptoms including depression and anxiety in post-COVID-19 patients (68). While these observations are promising for the potential efficacy of HBO_2_ in treating MDD, it is still unclear if it is the causative factor to the reductions in MDD symptoms, or if the improvements are due to confounding factors such as the alleviation of disease symptoms, or better nursing care. An improvement in symptoms of underlying conditions is likely to lead to a decrease in depressive feelings experienced, but the supportive effect of HBO_2_ in neuroplasticity and neurogenesis could imply that it has neurophysiological antidepressant effect as well (52).

Indeed, HBO_2_ studies that assess depressive symptoms as an outcome measure have consistently found depressive symptoms to improve after HBO_2_ treatment. Through animal and cellular models, HBO_2_ has demonstrated neuroprotective effects at multiple levels. At the cellular level, HBO_2_ improves mitochondrial metabolism and reduces cellular apoptosis, while also promoting neurogenesis through endogenous stem cells (69). Systemically, HBO_2_ exhibits anti-inflammatory effects, which could counteract the chronic neuroinflammation seen in MDD. Potential pathophysiological aspects of MDD that HBO_2_ could treat include neuroinflammation and neuronal atrophy, specifically kynurenine excitotoxicity, cortisol toxicity, and neuronal atrophy. While the findings are promising, our review is limited by the lack of studies assessing HBO_2_ in a sample population of patients with MDD as their primary diagnosis. As such, findings can only be categorized as indirect evidence, highlighting the need for future studies to examine the direct effect of HBO_2_ on MDD pathophysiology. Table 1 summarizes the HBO_2_ studies discussed in this section, including the treatment population, HBO_2_ protocol, outcomes, and limitations (Table 1).

**Table 1:** Summary table of available clinical studies investigating the therapeutic effect of HBO_2_ on neuropsychiatric and neurological conditions.

Study	Study design	Patient population	HBOT protocol	Outcomes	Limitations
Doenyas-Barak et al. (57)	RCT	Male veterans aged 25–60 years old with combat associated, treatment resistant PTSD.	60 sessions, 90 min, 100% oxygen at 2.0 ATA	Primary outcome: - DSM-V PTSD sclae score (CAPS-V) Secondary outcome: - Brief symptom inventory-18 (BSV-18) - Beck depression inventory-II (BDI-II) - Brain MRI diffusion tensor imaging (DTI) - n-back task in functional MRI (fMRI)	Findings on effect of HBOT on depressive symptoms cannot be interpreted as direct evidence for the therapeutic potential of HBOT for MDD. The study also only included male veterans, limiting the generalizability of results.
Harch et al. (61)	RCT	18–65 years old adults that have experienced at least one mild traumatic brain injury incident.	60 sessions, 90 min, 100% oxygen at 150 kPa (1.48 ATA)	Primary outcomes: - Neurobehavioral symptom inventory (NSI) - Wechsler Memory Scale-Version IV (WMS-IV) Secondary outcomes: - Post-Traumatic Stress Disorder Checklist Military or Civilian (PCL-M or -C 4) - Hamilton Depression Scale (HAM-D) - Hamilton Anxiety Scale (HAM-A) - Wechsler Adult Intelligence Scale-Version IV (WAIS-IV) - Rey Auditory Verbal Learning Test (RAVLT) - Benton Visual Retention Test - Stroop Color-Word Interference Test - Controlled Oral Word Association Test - Category Fluency Test - Automated Neuropsychological Assessment Metrics (ANAM-4) - Pittsburgh Sleep Quality Index (PSQI) - Quality of Life after Brain Injury (QOL)	Findings on effect of HBOT on depressive symptoms cannot be interpreted as direct evidence for the therapeutic potential of HBOT for MDD.
Feng et al. (63)	RCT	Patients aged 18–50 years old diagnosed with spinal cord injury and mild to moderate depression and/or anxiety.	48 sessions, 110 min each, 2.0 ATA % oxygen not specified.	Primary outcomes: - American Spinal Injury Association (ASIA) score - Functional independence measure (FIM) - Hamilton Depression Scale (HAM-D) - Hamilton Anxiety Scale (HAM-A)	Findings on effect of HBOT on depressive symptoms cannot be interpreted as direct evidence for the therapeutic potential of HBOT for MDD. The study also lacked an *a priori* power analysis to calculate a sufficient sample size, which could the results to be underpowered.
Sun et al. (65)	Retrospective Chart Review	Patients diagnosed with cerebral infarction for the first time with hemiplegia sequelae, stable pathology presentation, and a disease duration of greater or equal to 7 days.	Not provided	Primary outcomes: - Fugl-Meyer Assessment (FMA) - National Institutes of Health Stroke Scale (NIHSS) - Self-rating Anxiety/Depression Scale (SAS/SDS) Secondary outcomes: - Barthel index (BI) - Nursing effect	The study did not specify the HBOT protocol used. Participants in the treatment group received additional enhanced nursing care on top of HBOT compared to the control group, introducing confounding factors to the interpretation of results. Findings on effect of HBOT on depressive symptoms cannot be interpreted as direct evidence for the therapeutic potential of HBOT for MDD.
Guo et al. (66)	RCT	Patients aged 18 or above diagnosed with diabetes and post stroke depression	40 min each at 2.48 ATA. Specific number of sessions not specified, based on information provided number of sessions is estimated to be 30. % oxygen used not specified.	Primary outcomes: - Montgomery Depression Rating Scale (MADRS). - National Institutes of Health Stroke Scale (NIHSS) - Blood sample of TNF-α	The study did not specify the % oxygen and number of HBOT sessions administered. Findings on effect of HBOT on depressive symptoms cannot be interpreted as direct evidence for the therapeutic potential of HBOT for MDD.
Zilberman-Itskovich et al. (68)	RCT	Patients aged 18 or above with post-COVID-19 condition for at least 3 months aftger confirmed infection of COVID-19.	40 sessions, 90 min, 100% oxygen at 2.0 ATA.	Primary outcomes: - Mindstreams computerized cognitive testing battery Secondary outcomes: - Whole brain quantitative perfusion analysis - Diffusion brain volumes - short form-36 (SF-36) - Pittsburgh Sleep Quality Index (PSQI) - Brief Symptom Inventory (BSI-18) - Brief Pain Inventory (BPI) - Sniffin’ Sticks Test - Taste Strip Test - Pulmonary function measurement	Findings on effect of HBOT on depressive symptoms cannot be interpreted as direct evidence for the therapeutic potential of HBOT for MDD. Small sample size

### 5.3 HBO_2_ treatment protocol for PTSD and TBI

While there is currently no gold-standard dosage or treatment course for HBO_2_, protocols typically range from 40 to 60 sessions, with an atmosphere absolute (ATA) ranging from 1.3 to 2.0 ATA (70). Improvements in PTSD symptoms such as hyperarousal, depression, sleep disturbance, and intrusive symptoms were typically observed after HBO_2_ treatment, while side effects include middle ear barotrauma, sinus barotrauma, and rarely pulmonary barotrauma, seizures, and hypoglycemia (71). PTSD patients have reported a temporary worsening of symptoms during the course of HBO_2_ treatment, including emotional instability, depression, claustrophobia, fibromyalgia, and the resurfacing of traumatic memories (71). However, these changes were transient, and symptoms improved as the HBO_2_ treatment course progressed (71).

### 5.4 HBO_2_ dosing

As mentioned previously, although there lacks a standard recommended dosage for HBO_2_ treatment, prescription typically ranges from 40 to 60 sessions at 1.3–2.0 ATA. Multiple studies have seen an improvement in depressive and anxiety symptoms for patients undergoing the lower end of the 40–60 session range (61, 63). Decreasing dose treatment course duration may be beneficial as difficulties with adhering to the full HBO_2_ treatment course has been indicated as an issue in existing literature (57, 61). Thus, it may be productive for future studies to investigate if shorter HBO_2_ treatment courses can produce the same therapeutic effect for depressive symptoms. In addition to dosage considerations, longevity and duration of the treatment effect is also highly relevant when using HBO_2_ to treat conditions known for reoccurring, such as MDD. In an RCT on HBO_2_ use for long COVID, the authors found that participants who received HBO_2_ were able to maintain the acute neuropsychological improvements observed immediately post- HBO_2_ treatment for over a year (72). Similarly, in one of the HBO_2_ for PTSD studies mentioned previously, over 90% of participants reported their symptom improvements persisted after the initial HBO_2_ therapy (58). Findings in the literature provide rationale for investigating the efficacy of HBO_2_ at higher dose for a shorter treatment course duration, as well as the long-term retention of therapeutic effects from HBO_2_, particularly in patients with chronic illnesses such as PTSD.

## 6 Conclusions

In summary, literature in the area of HBO_2_ as a treatment for brain health is in its developmental phase, with existing studies focusing on conditions such as PTSD, PSD, and TBI. Understanding the multi-faceted mechanisms of action of HBO_2,_ our review highlights the intersections of these effects with the pathophysiology of MDD. However, while many HBO_2_ studies include investigations of changes in depressive symptoms as secondary outcomes, the observations provide at best, indirect evidence. There is a current dearth of studies that investigate the direct effect of HBO_2_ on patients with MDD. Given the limitations of existing treatment for MDD, there is a clear need to continue the development of new therapeutics and provide MDD patients with more treatment options that utilize novel mechanisms of action to address the substantial morbidity and mortality associated with this illness.
